# Synovial Sarcoma in the Extremity: Diversity of Imaging Features for Diagnosis and Prognosis

**DOI:** 10.3390/cancers15194860

**Published:** 2023-10-05

**Authors:** Eun Byul Cho, Seul Ki Lee, Jee-Young Kim, Yuri Kim

**Affiliations:** 1 Department of Radiology, Uijeongbu St. Mary’s Hospital, College of Medicine, The Catholic University of Korea, Uijeongbu 11765, Republic of Korea; 2 Department of Radiology, St. Vincent’s Hospital, College of Medicine, The Catholic University of Korea, Seoul 06591, Republic of Korea

**Keywords:** synovial sarcoma, imaging spectrum, CT, MRI, diagnosis, prognosis

## Abstract

**Simple Summary:**

Synovial sarcomas are the fourth most prevalent soft-tissue sarcoma, primarily affecting the extremities (in 80–95% of cases) of adolescents and young adults aged 15 to 40. The imaging characteristics of synovial sarcomas encompass a wide spectrum, ranging from non-aggressive features to more aggressive appearances. We have conducted a comprehensive review, illustration, and correlation of the radiological features of synovial sarcomas, aiming to assist in their diagnosis and prognosis. Understanding and identifying these diverse presentations, along with their associated prognostic implications, might be helpful in patient evaluations and in achieving optimal management.

**Abstract:**

Synovial sarcomas are rare and highly aggressive soft-tissue sarcomas, primarily affecting adolescents and young adults aged 15–40 years. These tumors typically arise in the deep soft tissues, often near the large joints of the extremities. While the radiological features of these tumors are not definitely indicative, the presence of calcification in a soft-tissue mass (occurring in 30% of cases), adjacent to a joint, strongly suggests the diagnosis. Cross-sectional imaging characteristics play a crucial role in diagnosing synovial sarcomas. They often reveal significant characteristics such as multilobulation and pronounced heterogeneity (forming the “triple sign”), in addition to features like hemorrhage and fluid–fluid levels with septa (resulting in the “bowl of grapes” appearance). Nevertheless, the existence of non-aggressive features, such as gradual growth (with an average time to diagnosis of 2–4 years) and small size (initially measuring < 5 cm) with well-defined margins, can lead to an initial misclassification as a benign lesion. Larger size, older age, and higher tumor grade have been established as adverse predictive indicators for both local disease recurrence and the occurrence of metastasis. Recently, the prognostic importance of CT and MRI characteristics for synovial sarcomas was elucidated. These include factors like the absence of calcification, the presence of cystic components, hemorrhage, the bowl of grape sign, the triple sign, and intercompartmental extension. Wide surgical excision remains the established approach for definitive treatment. Gaining insight into and identifying the diverse range of presentations of synovial sarcomas, which correlate with the prognosis, might be helpful in achieving the optimal patient management.

## 1. Introduction

Synovial sarcomas, which are malignant soft tissue tumors, are believed to constitute 5–10% of all soft tissue sarcomas, standing as the most common malignancy of the lower extremity among individuals aged 15 to 40 years [1,2,3]. Following rhabdomyosarcoma, synovial sarcomas rank as the second most prevalent type of soft-tissue sarcoma among children, adolescents, and young adults [1,4,5]. According to data from the SEER (Surveillance, Epidemiology, and End Results) database, synovial sarcomas are primarily diagnosed during the third decade of life, with only a small portion (2.5%) of cases occurring in children under the age of 10 [6]. Younger children have more favorable outcomes, compared to adults and adolescents [1]. Both males and females seem to be equally affected [7]. 

Approximately 80% to 95% of synovial sarcomas manifest in the extremities, with the intrathoracic and head and neck regions being less frequently affected [8,9]. Synovial sarcomas in the extremities tend to appear as slowly enlarging masses, occasionally accompanied by pain, and often go unnoticed until a triggering event such as trauma occurs [10]. Intrathoracic synovial sarcomas have potential to cause chest pain and shortness of breath, while synovial sarcomas in the head and neck regions may exhibit symptoms like hoarseness, odynophagia, or epistaxis [11,12,13,14,15]. Synovial sarcomas can arise in various locations within the body, leading to diverse clinical presentations depending on their location [16]. These tumors can display an indolent nature, characterized by a seemingly gradual growth rate, and they frequently appear small in size (<5 cm) upon initial detection, often with well-defined margins [9]. A majority of patients present with a lengthy history of a slowly growing lump, spanning an average duration of 2 to 4 years before seeking medical attention [9]. 

The gradual rate of growth can give rise to a deceptive perception of benignity, which could potentially lead to delays in the diagnostic process [9]. However, synovial sarcomas display aggressive behavior, evidenced by local recurrence rates up to 50%, with as many as a quarter of patients developing distant metastases. The lung and bone are the most frequent sites of metastases. Five-year survival rates range from 24% to 71%, influenced by a range of prognostic factors, including age, location, and histological grades [17]. These tumors are categorized as moderate- to high-grade lesions (II/III) in terms of histology [18]. Synovial sarcomas are classified into three subtypes based on histologic characteristics: monophasic, biphasic, or poorly differentiated [9]. The monophasic subtype, composed solely of spindle cells, is the most prevalent. The biphasic subtype contains both spindle cells and epithelial cells. Lastly, the poorly differentiated subtype is composed of cells resembling those found in small round blue cell tumors [19]. Despite the shared genetic and pathophysiological features, a distinct difference in outcomes between pediatric and adult patients is well-established, with a higher 5-year survival rate in children (83–89%) compared to adults (43–76%) [1,20,21].

Contrary to their name, these tumors do not originate from synovial cells. Instead, they are categorized as mesenchymal spindle cell tumors displaying varying degrees of epithelial differentiation. They are identified by a specific chromosomal translocation involving SS18 and SSX1, resulting in the formation of the SS18 SSX fusion oncogene [22]. This fusion oncogene leads to the disruption of multipotent mesenchymal stem cells, which are considered to be the cellular origin of synovial sarcomas [23]. The majority of these tumors (80–95%) are found in the extremities, with around two-thirds situated in the lower limbs, often in close proximity to the knee. They are closely associated with the tendon sheath and bursae [24]. Despite originating predominantly in the musculoskeletal system, synovial sarcomas do not actually arise from synovium and are rarely found within the intra-articular space [25]. 

Due to their rarity, many clinicians and radiologists are often unfamiliar with the presentation and imaging characteristics of synovial sarcomas. It is crucial that interpreting radiologists or orthopedic oncologists avoid categorizing these tumors as benign. Erroneously assuming the benign nature of synovial sarcomas can result in diagnostic delays and potentially impact prognosis [26]. Notably, an early diagnosis of smaller lesions holds significance for managing local disease, as complete surgical resection is associated with improved survival [27]. The objective of this review article is to outline the diverse range of imaging features observed in extremity synovial sarcomas, spanning from non-aggressive to aggressive appearance. The intention is to facilitate accurate diagnosis and characterization through preoperative imaging, emphasizing the prognostic value of these imaging features for extremity synovial sarcomas.

## 2. Diagnostic Imaging Features of the Extremity Synovial Sarcoma 

### 2.1. Radiographs

**Figure 1:**
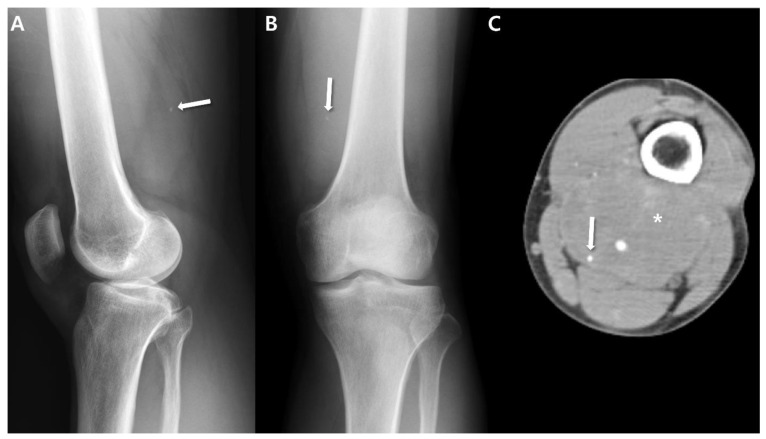
Stippled calcification in synovial sarcoma of 28-year-old man with synovial sarcoma (monophasic type): (**A**) Lateral radiograph of knee shows stippled calcification (arrow) in soft tissue opacity of distal femur. (**B**) Anteroposterior radiograph also shows stippled calcification (arrow) in soft tissue opacity. (**C**) Lower extremity CT angiography shows stippled calcification (arrow) in heterogenous enhancing mass (*) of distal femur.

Conventional radiograph is frequently the initial study conducted, potentially revealing indications of a soft-tissue mass through abnormal contouring of the skin, displacement effects on native structures, or heightened soft tissue density [28]. The American College of Radiology Appropriateness Committee continues to recommend conventional radiography, even for superficial masses, as a valuable diagnostic method for assessing mineralization within the mass and bone erosion [29]. In up to 30% of synovial sarcomas, calcifications may also be detectable and their presence should elevate suspicion toward the possibly of a synovial sarcoma (Figure 1) [9,17,30]. When observed, these calcifications often exhibit an eccentric or peripheral arrangement within the soft-tissue mass [9]. Occasionally, cases of synovial sarcoma with calcification can resemble osteosarcomas or other conditions linked to calcifications [9]. Wilkerson et al. [30] investigated the calcification patterns of synovial sarcomas, encompassing twenty-four monophasic types and five biphasic types. Among these cases, 58.6% (17/29) displayed calcification in imaging; nine cases exhibited scattered stippled calcifications (Figure 1) and eight cases featured stippled calcifications in clusters. Two cases further exhibited additional irregular spicules of calcifications. The researchers concluded that fine, stippled calcifications are atypical in other malignant soft tissue tumors but are characteristic of synovial sarcoma; the presence of such calcifications in a soft-tissue mass in preoperative images, particularly in a younger patient, should strongly prompt consideration of synovial sarcoma as the potential diagnosis [30]. 

### 2.2. Computed Tomography (CT) 

**Figure 2:**
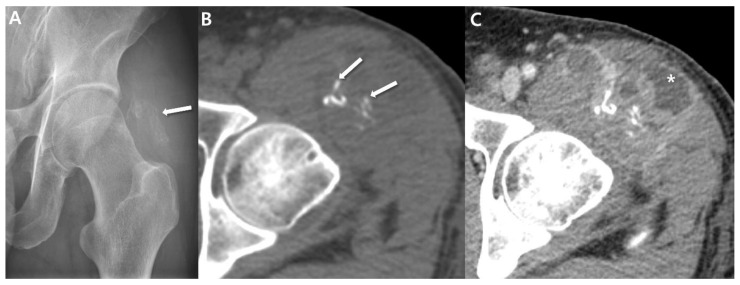
CT features of synovial sarcoma of 54-year-old man (undifferentiated type) containing coarse calcifications: (**A**) Pelvis outlet radiograph shows coarse calcifications (arrow) in soft tissue opacity of Lt upper thigh. (**B**) Axial precontrast CT shows coarse calcifications (arrows) in large mass with heterogenous in density. (**C**) Axial post-contrast CT shows heterogeneously enhancing mass including non-enhancing region (*), suggesting necrotic or cystic change.

The prevalent CT presentation of synovial sarcomas typically displays a heterogeneous deep-seated, soft-tissue mass with attenuation similar to or slightly lower than that of muscle [9]. Regions of lower attenuation, indicating necrosis or hemorrhage, are also frequently observable (Figure 2), although smaller lesions may exhibit a more uniform appearance [9]. Tateishi at el. [31] documented that a well-defined margin was evident in 53% of cases, whereas 47% displayed an irregular margin. Subsequent to the administration of intravenous contrast agents, the mass demonstrates heterogeneous enhancement in 89–100% of cases (Figure 2) [31]. This characteristic is notably valuable in distinguishing synovial sarcomas that might initially resemble cystic lesions or hematomas on precontrast images [32].

CT also proves valuable in the identification of subtle soft-tissue calcifications within synovial sarcoma and local bony changes (either as erosion or bone marrow invasion), particularly in anatomically complex regions like the pelvis, hip, or shoulder [9]. Wilkerson et al. [30] assessed the patterns of calcification in synovial sarcomas: (i) Fine and stippled—characterized as fine, punctate, and smaller than 2 mm (Figure 1); (ii) Coarse spicules—presenting as irregularly shaped, branching, and larger than 3 mm (Figure 2). The researchers speculated that the varying calcification appearances in synovial sarcomas mirror different stages in maturation; finer calcifications are observed earlier in the tumor’s development compared to the appearance of coarser calcifications [30]. Bone involvement has been observed in approximately 25% of cases in a previous study [9].

### 2.3. Ultrasound

**Figure 3:**
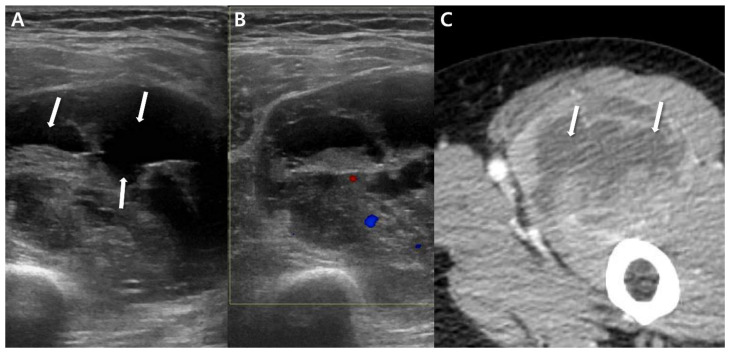
Ultrasonographic features of synovial sarcomas of 63-year-old women (undifferentiated type): (**A**) Ultrasound shows heterogeneously anechoic and echogenic mass. Anechoic regions (arrows) reflecting cystic/necrotic change. (**B**) Doppler image shows tumor vascularity. (**C**) Axial post-contrast CT image shows heterogeneously enhancing mass including non-enhancing portion (arrows) in left thigh.

Ultrasounds typically serve as the primary investigative method for assessing soft-tissue masses, primarily employed to discern between cystic and solid lesions. Small tumors (less than 5 cm) generally exhibit an ovoid shape, slight lobulation, and a homogenous echotexture, similar in echogenicity to muscle [33]. In larger masses, a significant degree of heterogeneity with irregular margins has been observed (Figure 3) [33]. Hypo- or anechoic areas reflect cystic or necrotic change, while more heterogeneously echoic regions correspond to cellular areas of viable tumor and hyperechoic regions indicate occurrences of hemorrhage, calcification, or fibrosis [9]. In cases of aggressive tumors, surrounding edema might be visible [17]. Doppler interrogation commonly reveals vascularity (Figure 3) [9].

### 2.4. Magnetic Resonance Imaging (MRI) 

MRI stands as the favored imaging technique for delineating the nature of soft tissue sarcomas and establishing the tumor’s extent prior to surgery [34]. MRI protocols must include at least the following sequences: one T1-weighted sequence prior to the administration of gadolinium chelate, one T2-weighted sequence with or without fat-suppression techniques (such as fat-saturation, fluid-sensitive, or short tau inversion-recovery sequences), and one T1-weighted sequence following the injection of gadolinium chelate, with fat suppression (including the DIXON method or subtraction with precontrast imaging) [35]. Synovial sarcomas are most effectively assessed using MRI, given its superior capability to distinguish soft tissues. The MRI presentations of synovial sarcomas encompass a spectrum, spanning from small, uniform nodules to extensive, heterogeneous masses [17,36]. 

**Figure 4:**
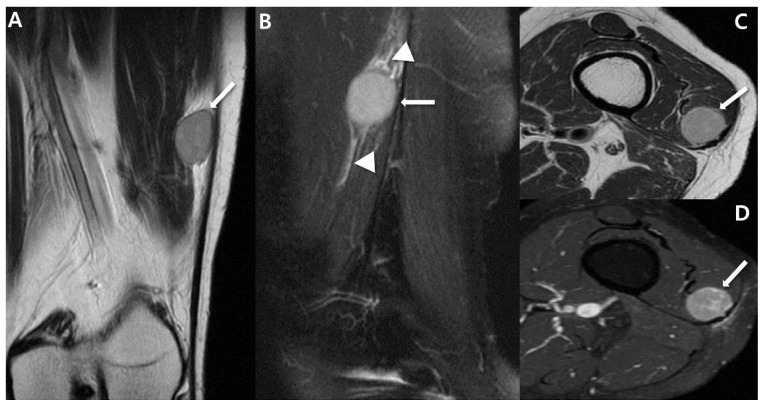
Non-aggressive features on MRI for small (<5 cm) synovial sarcoma of 42-year-old women (monophasic type): (**A**) Coronal T2-weighted image shows about 1.6 × 1.6 × 2.3 cm sized well-defined oval mass (arrow) with homogenous and relatively high signal intensity compared to adjacent muscle in the thigh. (**B**) Sagittal T2-weighted, fat-suppressed image shows a mass (arrow) abutting vastus lateralis muscle (arrowheads) with adjacent signal change. (**C**) Axial T2-weighted image shows well-defined oval mass (arrow) located in vastus lateralis muscle. (**D**) Axial T1-weighted post-contrast image shows relatively homogeneously enhancing mass (arrow).

**Figure 5:**
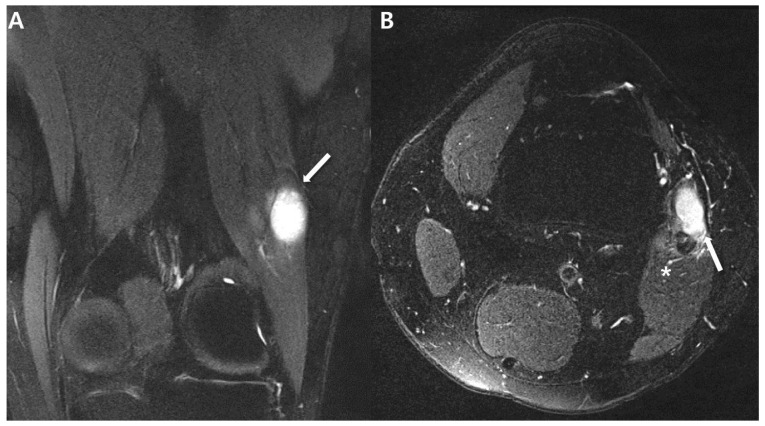
Non-aggressive features on MRI for small (<5 cm) synovial sarcoma of 22-year-old men (biphasic type): (**A**) Coronal T2-weighted, fat-suppressed image shows about 1.2 × 1.7 × 1.9 cm sized lobulated mass (arrow) in the lateral aspect of Lt knee. (**B**) Axial T2-weighted. fat-suppressed image shows a mass (arrow) with homogenously bright signal intensity, abutting biceps femoris (*) with adjacent signal change.

Black sin et al. [37] examined 15 patients diagnosed with histologically confirmed synovial sarcomas, revealing that smaller lesions measuring ≤5 cm might exhibit a non-aggressive appearance characterized by well-defined margins and homogeneous signal intensity (SI). Small synovial sarcomas can present as lesions with well-circumscribed and homogenous hyperintensity on fluid-sensitive sequences (Figure 4) or even as totally cystic lesions (Figure 5), which might mimic benign conditions such as ganglion cysts or bursae [17]. These tumors have the potential to be mistaken for benign lesions, leading to inappropriate surgical interventions like excisional biopsies performed via transverse incisions [37]. 

**Figure 6:**
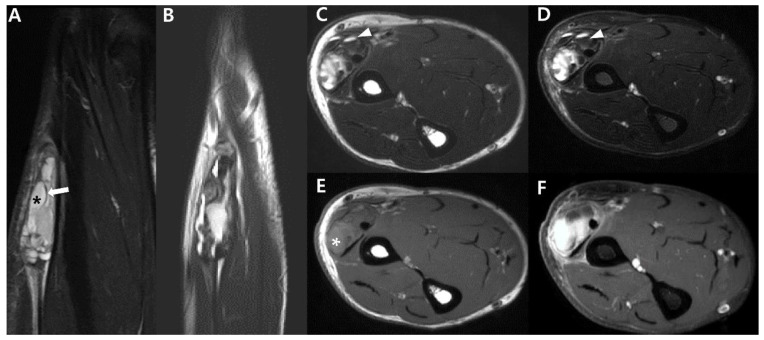
Aggressive features on MRI for large heterogenous mass of synovial sarcoma of 61-year-old-man (monophasic type): (**A**) Coronal T2-weighted, fat-suppressed image shows about 1.5 × 2.3 × 7.0 cm sized multilobulated mass with heterogenous signal intensity of Lt forearm. The mass shows presence of T2 hyperintense areas (*) with intervening T2 hypointense septa (arrow), called ‘bowl of grapes’. (**B**) Sagittal T2-weighted image also shows heterogenous intensity. (**C**,**D**) Axial T2-weighted images without and with fat suppression show well-circumscribed heterogenous signal intensity mass with fluid–fluid level (arrowheads), suggesting presence of hemorrhage. (**E**) Axial T1-weighted image shows iso to high (*) signal intensity compared to adjacent muscle. High signal intensity region suggests hemorrhage component. (**F**) Axial T1-weighted, post-contrast image shows heterogeneous enhancement.

**Figure 7:**
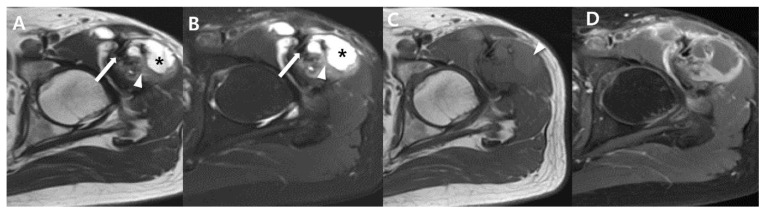
Aggressive features on MRI for large heterogenous mass of synovial sarcoma of 54-year-old-man (undifferentiated type; same patient with Figure 2): (**A**,**B**) Axial T2-weighted images without and with fat suppression show about 8.5 × 5.4 × 9.2 cm sized ill-defined heterogenous signal intensity mass including low (arrow), iso (arrowhead), and high (*) signal intensity compared to adjacent muscle, called ‘triple sign’ in left rectus femoris muscle. (**C**) Axial T1-weighted image shows iso to high signal intensity compared to adjacent muscle. High signal intensity region suggests hemorrhage component (arrowhead). (**D**) Axial T1-weighted, post-contrast image shows heterogeneous enhancement.

**Figure 8:**
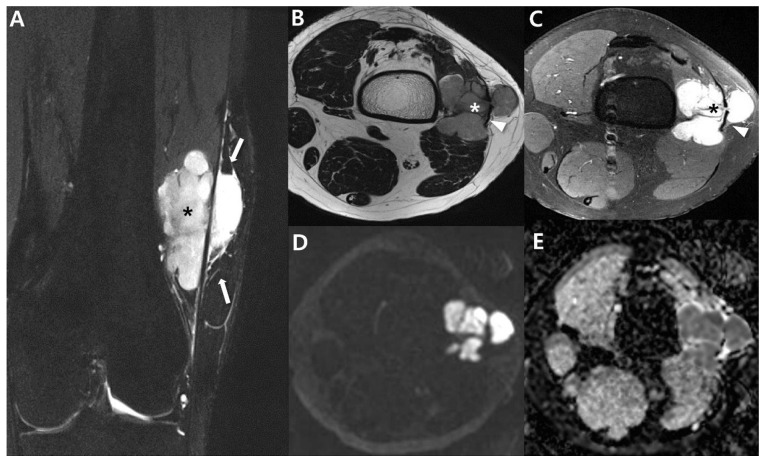
Aggressive features on MRI for large mass of synovial sarcoma of 22-year-old-man (biphasic type): (**A**) Coronal T2-weighted, fat-suppressed image shows about 3.5 × 3.9 × 5.2 cm sized multilobulated mass with heterogenous signal intensity of Lt vastus lateralis, focally involving biceps femoris muscle. The mass shows presence of T2 hyperintense areas (*) with interfascial (or intercompartmental) extensions (arrows). (**B**) Axial T2-weighted images show multilobulated, high-signal-intensity mass (*) with extension to subcutaneous fat layer through iliotibial band (arrowhead). (**C**) Axial T1-weighted, post-contrast image shows well-enhancing mass (*) with intercompartmental extensions (arrowhead). (**D**,**E**) Axial DWl (*b* = 1400) and ADC map show high signal intensity and low ADC value, suggesting diffusion restriction.

In larger masses (>5 cm), MRI typically reveals more heterogeneous signal intensity (SI) [37]. Most synovial sarcomas manifest as prominently heterogeneous, multilobulated soft-tissue masses, displaying SI similar to or slightly higher than that of muscle on T1-weighted images and predominantly bright SI on T2-weighted images [9,28]. However, around one-third of larger lesions exhibit distinctive features of heterogeneity on T2-weighted images, including the “bowl of grapes” appearance, indicating a multicystic mass with varying fluid intensities and “fluid–fluid levels”, as well as the “triple sign”, signifying areas of hyperintensity, isointensity, and hypointensity relative to skeletal muscle [17]. The “bowl of grapes” appearance may arise due to the presence of hyperintense areas on fluid-sensitive sequences with interspersed T2 hypointense septa (Figure 6) [38,39,40]. This finding is not unique to synovial sarcoma and is also seen in undifferentiated pleomorphic sarcoma [41]. “Fluid–fluid levels” can emerge due to the accumulation of sedimented blood products (Figure 6) [10], and these levels become visible when imaging is conducted in a gravity-dependent plane [31]. However, this finding can also be present in other soft-tissue masses such as hemangioma or undifferentiated pleomorphic sarcoma [42]. The “triple sign” is attributed to necrotic, cystic, or hemorrhagic areas (hyperintensity); cellular elements (isointensity); and calcified or fibrotic regions or areas of old blood (hypointensity) (Figure 7) [10]. Nonetheless, this sign is also observable in other soft-tissue tumors, especially undifferentiated pleomorphic sarcoma [9]. Aggressive tumors often display peritumoral edema [17]. Moreover, interfascial or intercompartmental extensions (Figure 8) frequently indicate the aggressiveness of the tumor [10]. 

Table 1 summarizes the imaging spectrum of synovial sarcomas.

### 2.5. Advanced MRI 

Advanced MRI techniques, including diffusion weighted imaging (DWI) and dynamic contrast enhanced imaging (DCE), offer the potential for enhanced precision in imaging-based diagnoses prior to the histopathologic confirmation [43,44,45,46,47]. The hypercellularity of malignant tumors results in restricted diffusion, evident in a lower apparent diffusion coefficient (ADC) value [47]. Tumor-associated angiogenesis and hypervascularity contribute to prompt arterial enhancement, detectable through DCE [45]. 

The Ashikyan study [43] evaluated advanced MRI sequences on a homogeneous sample of synovial sarcomas. The average minimum ADC was 0.8 × 10^−3^ mm^2^/s (standard deviation [SD] = 0.4 × 10^−3^ mm^2^/s), the average mean ADC was 1.2 × 10^−3^ mm^2^/s (SD = 0.5 × 10^−3^ mm^2^/s), and the average maximum ADC was 1.9 × 10^−3^ mm^2^/s (SD = 0.7 × 10^−3^ mm^2^/s) [43]. Synovial sarcomas exhibit heterogeneity with marked restricted diffusion (Figure 8). Lower ADC values are indicative of malignancy [43,48] and are found to correspond with higher grades of sarcoma [43,49]. In DCE MRI, the tumor blush became visible within 6–20 s after the arteries became visible. Progressive enhancement was observed with mild qualitative washout during the 3 min scanning period. Tumor vascularity was evident in all cases [43]. The average relative enhancement of tumors was 5.7 times that of the adjacent skeletal muscle, with a minimum enhancement of 1.5 times and maximum enhancement of 17.6 times (SD of 4.9 times) [43]. Synovial sarcomas display pronounced early contrast enhancement in DCE MRI [43]. 

Utilizing advanced MRI sequences prior to selecting biopsy sites is crucial for obtaining more accurate information and minimizing sampling errors, as synovial sarcomas exhibit structural heterogeneity with the presence of necrotic and fluid-filled components [43]. Furthermore, these investigations exhibit strong correlations with synovial sarcoma grading, enabling predictions regarding tumor margin infiltration, local recurrence, or adverse prognosis [50,51,52,53,54,55]. 

## 3. Uncommon Primary Sites of Synovial Sarcoma in the Extremities 

**Figure 9:**
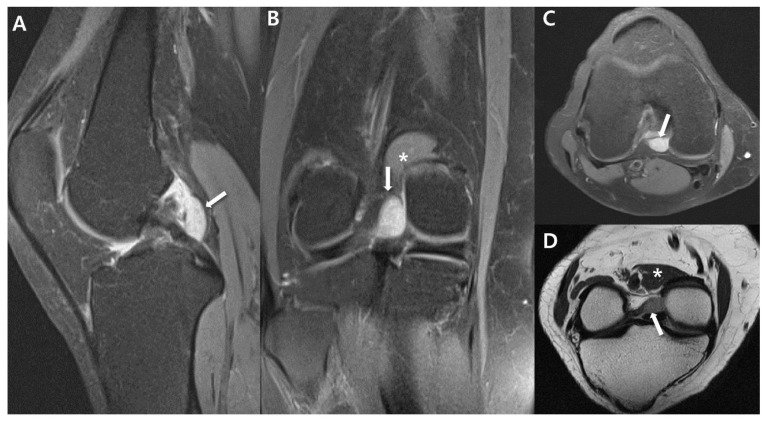
Intra-articular synovial sarcoma of 44-year-old-women (monophasic type): (**A**) Sagittal proton-weighted, fat-suppressed image shows about 2.0 × 1.2 × 3.0 cm sized circumscribed oval nodule (arrow) around posterior femoral recess of left knee. (**B**,**C**) Coronal and axial proton-weighted, fat-suppressed images show homogenous high signal intensity nodule (arrow) compared to adjacent muscle (*). (**D**) Axial oblique T2-weighted image shows a nodule (arrow) with relatively high signal intensity compared to muscle (*) but not bright high signal intensity such as ganglion cyst.

Synovial sarcomas represent the most prevalent soft tissue malignancy in the age range of 15 to 40 years, primarily affecting the lower extremity. These tumors can develop within the skeletal muscle and the supporting connective tissue of the limbs [56]. Despite their name, primary intra-articular synovial sarcomas are extremely rare [56]. The precise incidence of synovial sarcomas arising within a joint remains uncertain; however, considering the scarcity of published case reports, it is widely believed to be quite infrequent. Less than 5% of synovial sarcomas exhibit a connection with the synovium [57,58]. Distinguishing these tumors can be challenging, as they might be misdiagnosed as benign intra-articular conditions such as ganglion cysts, tenosynovial giant cell tumors, synovial osteochondromatosis, or synovial hemangiomas [59]. While the appearance of intra-articular synovial sarcomas on MRI lack specificity, they should be taken into consideration when encountering an intra-articular mass, especially within the knee joint. This is particularly relevant if the mass does not exhibit the characteristic radiological features of common benign intra-articular diseases like ganglion cysts, tenosynovial giant cell tumors, synovial osteochondromatosis, or synovial hemangiomas [59]. For instance, even in cases where the lesion looks like an intra-articular ganglion cyst, suspicion of synovial sarcoma should arise if the brightness on T2-weighted images is either only slightly elevated or not significantly intense, or if there is evidence of calcification (Figure 9). Administration of intravenous contrast material can aid in distinguishing between a ganglion cyst and the synovial sarcoma. In particular, synovial sarcoma tends to display inhomogeneous enhancement, whereas ganglion cysts typically remain unenhanced [60].

**Figure 10:**
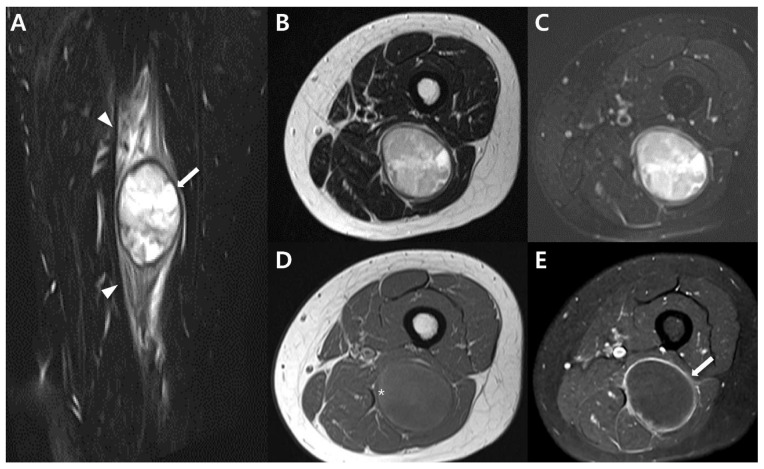
Intraneural synovial sarcoma of 37-year-old-women (monophasic type): (**A**) Coronal T2-weighted, fat-suppressed image shows about 4.3 × 4 cm sized oval circumscribed mass (arrow) along the sciatic nerve in posterior aspect of Lt thigh. Note extensive peritumoral edema (arrowheads). (**B**,**C**) Axial T2-weighted images without and with fat suppression show well-circumscribed mass between semimembranosus and semitendinosus muscle. The mass shows heterogenous signal intensity without typical target sign. (**D**) Axial T1-weighted image shows iso to high (*) signal intensity compared to adjacent muscle. High (*) signal intensity region suggests hemorrhage component. (**E**) Axial T1-weighted post-contrast image shows peripheral rim enhancement (arrow), suggesting totally cystic change.

Masses originating from peripheral nerves and nerve sheaths are frequently encountered, with the majority being attributed to benign peripheral nerve sheath tumors (BPNST) like schwannomas (neurilemmomas) and neurofibromas [61,62]. However, peripheral non-neural sheath tumors are rare, except for the more prevalent ganglion cysts of the peripheral nerves [63]. Synovial sarcoma arising within a peripheral nerve stands as an exceedingly rare occurrence, with only 39 published cases to date. Most of these cases are reported individually, although the most extensive series encompassed ten patients [64,65,66]. These tumors can pose diagnostic challenges in terms of diagnosis, as their imaging characteristics can closely resemble those of BPNSTs, potentially leading to unplanned excision [65,67]. On imaging, synovial sarcomas of the peripheral nerves can imitate BPNSTs, and making a clear distinction between the two based solely on imaging features can prove to be exceedingly difficult [64]. Larque et al. [64] presented a case series of synovial sarcomas of the nerve, noting that this condition usually manifests as a well-defined mass on imaging, similar to BPNST. Nevertheless, the presence of atypical features, such as pain, larger size, lack of target sign, irregular margins, or the presence of peritumoral edema, could aid in distinguishing synovial sarcoma from other conditions (Figure 10) [64,68].

## 4. Prognostic Imaging Features of Synovial Sarcoma in the Extremity

Up to 50% of synovial sarcomas exhibit local recurrence within two years and between 48% to 68% of patients experience the development of distant metastases, with 6 to 14% of these cases manifesting at the time of initial diagnosis [8,10,14]. The 5-year survival rate for synovial sarcomas ranges from 66% to 71% [8]. Notably, it is important to acknowledge that delayed local recurrences and metastases occurring more than 5 years after the initial diagnosis are common, which might necessitate extended follow-up periods [39]. Numerous clinical and pathological prognostic factors have been documented. These include factors like patient age, tumor size, tumor grade, histologic subtypes, anatomical location, the effectiveness of local treatments with negative surgical margins, and the use of additional treatments such adjuvant radiotherapy [22,69,70,71,72,73,74,75]. Notably, patient under the age of 15 to 20 years tend to have a more favorable long-term prognosis [76]. Among these factors, the size of the tumor at presentation, particularly tumors larger than 5 cm, significantly influences prognosis. Studies indicate that 5-year survival rates are 64% for patients with tumors smaller than 5 cm but only 26% for those with masses exceeding 5 cm [8]. The prognosis also varies depending on the location of the tumor. Patients with tumors situated in the extremities generally have a more favorable prognosis compared to those with tumors in the head and neck region or axial region [9]. This discrepancy likely arises from the better surgical control available for lesions in the extremities. Notably, Deshmukh et al. [77] reported poorer prognosis for patients with synovial sarcomas located in the proximal extremities. Recent investigations have explored the prognostic significance of imaging features observed on CT and MRI for synovial sarcomas [5,10,17,31]. These studies have aimed to better understand the correlations between imaging characteristics and prognosis. 

### 4.1. Metastatic Pattern

**Figure 11:**
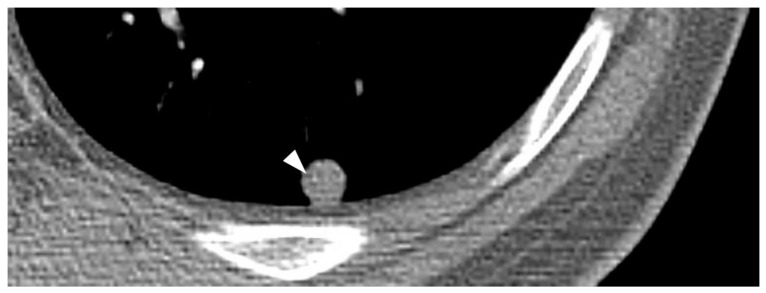
Pulmonary metastasis from synovial sarcoma containing tiny calcification. Precontrast CT image shows small metastatic synovial sarcoma in the lung and this nodule contains tiny calcification (arrowhead).

**Figure 12:**
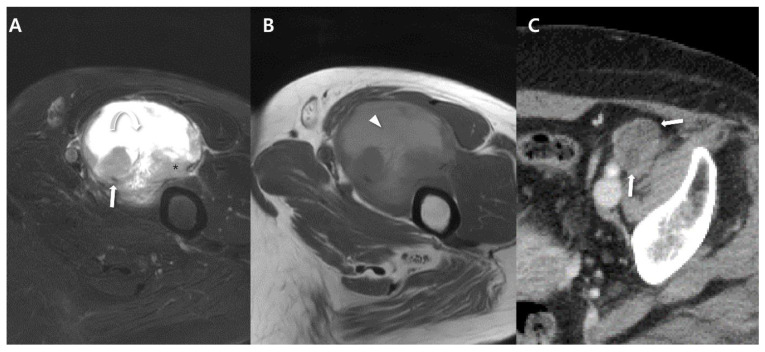
Lymph node metastasis from synovial sarcoma (same patient with Figure 3): (**A**,**B**) Initial MRI of axial T2-weighed, fat-suppressed and T1-weighted images shows about 6.3 cm sized soft-tissue mass with triple sign (curved arrow: hyperintense, *: isointense, arrow: hypointense) and hemorrhagic change (arrowhead). (**C**) Follow-up CT after 1 year shows regional lymph node metastasis (arrows).

**Figure 13:**
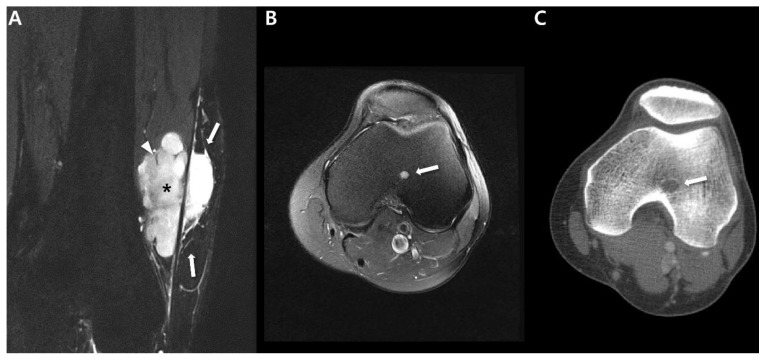
Bone metastasis from synovial sarcoma (same patient with Figure 8). (**A**) Initial MRI of coronal T2-weighed, fat-suppressed show about 5.2 cm sized soft-tissue mass (*) with intervening septa (arrowhead) showing ‘bowl of grapes’ and intercompartmental extensions (arrows). (**B**) Follow-up MRI of axial T2-weighted, fat-suppressed image after 3 years shows small round bone metastasis (arrow). (**C**) Metastatic lesion shows osteolytic appearance (arrow) on CT.

Synovial sarcomas exhibit a notable propensity for metastatic disease, with various studies reporting distant metastases developing in 48% to 68% of patients [8,14,18]. According to Baheti et al. [10], who conducted a study on this matter, the most common sites of metastases were the pleuropulmonary regions, lymph nodes, and bones. An important observation from Baheti’s study is that the intrathoracic metastatic disease frequently manifested as pleural-based involvement [10]. Notably, similar to primary lesions, metastatic lesions in synovial sarcomas often demonstrate calcification, which holds significance from an imaging perspective (Figure 11) [19]. Unlike most other soft-tissue sarcomas, synovial sarcomas can also give rise to nodal metastases, occurring in 8% to 27% of patients. Lymph node metastases are usually a later aspect of distant metastatic progression, particularly within the thorax (associated with pleuropulmonary metastases). Regional lymph node metastases can also occur alongside this (Figure 12) [10]. Additionally, certain other sarcoma types such as clear cell sarcoma, angiosarcoma, epithelioid sarcoma, alveolar soft-part sarcoma and rhabdomyosarcoma also exhibit a tendency for nodal metastases. These tumors can likewise spread to regional lymph nodes [10]. Regarding bone metastasis, Baheti’s findings revealed that such occurrences predominantly presented as lytic lesions (Figure 13) [10].

### 4.2. Prognostic Imaging Features on CT and MRI 

Several imaging characteristics associated with synovial sarcomas have been linked to prognosis. Generally, larger tumors located more proximally and exhibiting greater complexity have correlated with worse outcomes [37,76]. In the study by Tateishi et al. [31], specific locations such as the upper thigh, inguinal region, head and neck, and trunk, along with tumor size exceeding 5 cm (Figure 12 and Figure 13), absence of calcification, presence of intratumoral hemorrhage (Figure 12), and the manifestation of the triple sign (Figure 12) were all significantly connected with worse disease-free survival. Through multiple logistic regression models, it was established that tumor size larger than 10 cm exerted a noteworthy impact on the disease-free survival [31]. According to Baheti et al. [10], factors including tumor size, the presence of peritumoral edema, intercompartmental extension (Figure 13), intratumoral hemorrhage, and a bowl-of-grapes appearance (Figure 13) were significantly associated with higher rates of metastatic disease. However, these factors did not exhibit correlations with disease-free or overall survival. Interestingly, a prior study that differentiated between low-grade versus high-grade soft-tissue neoplasms observed that peritumoral edema was present in 80% of low grade tumors and 96% of high grade tumors [43]. In a recent study including nine patients with synovial sarcomas, they found that the most significant indicator of tumor grade (II–III) was the existence of peritumoral enhancement [34]. The presence of peritumoral enhancement, without causing any discernible mass effects on the neighboring tissues, is regarded as a direct sign of tumor margin infiltration [78]. 

A more favorable prognosis, on the other hand, is linked to extensive calcification and tumor size below 5 cm [31,79]. Notably, Baheti et al. found that none of the six patients with calcifications in their study had expired [10]. Additionally, Varela-Duran and Enzinger [80] also reported that the presence of extensive calcifications is indicative of improved long-term survival, with 5-year survival rates reaching 82%. Furthermore, the occurrence of local recurrence (32%) and metastatic disease (29%) was notably reduced in cases featuring extensive calcifications. Also, Mickael et al. [81] studied the subgroup of patients with synovial sarcomas (n = 34) with available CT scans; calcifications were associated with longer overall survival, with a median survival of 84.9 months (calcified synovial sarcoma) compared to 55.2 months (non-calcified synovial sarcoma). They concluded the presence of calcifications on CT scans is associated with favorable outcomes.

Table 2 summarizes the prognostic factors for synovial sarcomas.

## 5. Treatments and Management

The treatment for synovial sarcomas typically comprises a combination of surgery, radiotherapy, and chemotherapy. The specific approach is determined by factors such as the size and extent of the primary tumor, as well as the presence of distant metastases [9]. 

### 5.1. Surgical Treatment

Similar to other soft tissue tumors, surgery serves as the primary method for achieving local control in synovial sarcomas. The objective is to achieve wide surgical margins, which involves removing the entire tumor along with the affected anatomical compartment. Whenever feasible, efforts are made to employ limb-sparing surgery [9]. Attaining negative margins holds exceptional significance as it substantially decreases the likelihood of local recurrence and contributes to the overall survival rate [82]. Precise guidelines concerning the optimal margin dimensions for resecting tissue around the tumor mass in synovial sarcoma are not established. The mainstay of treatment for synovial sarcomas remains surgical excision with negative margins with potential augmentation through radiotherapy and/or chemotherapy, contingent upon the specific features of the patient and tumor [83]. 

### 5.2. Radiation Therapy 

Utilizing neoadjuvant or adjuvant radiation therapy has demonstrated its efficacy in enhancing local control when addressing synovial sarcomas, particularly for larger tumors (>5 cm), or situations where maintaining a narrow margin becomes essential to preserve crucial neurovascular structures or bones [83,84,85,86]. Additionally, radiation therapy is employed to ensure local control and has proven effective in reducing the decrease recurrence [17]. 

### 5.3. Chemotherapy

Synovial sarcomas are relatively chemosensitive when compared with other soft tissue sarcomas [17]. Unlike many other sarcomas, certain subgroups of synovial sarcomas appear to hold potential advantages from systemic therapy involving cytotoxic chemical agents [82,87,88]. Typically, chemotherapy is preserved for cases involving high-risk, large, and deep tumors that carry an approximately 50% likelihood of developing metastatic lesions. 

### 5.4. Novel Therapeutic Options

Considerable interest has arisen in the advancement of targeted medical therapies for managing synovial sarcoma [89,90]. Emerging evidence underscores the pivotal role of angiogenesis in the progression of soft tissue sarcomas, thereby indicating the potential utility of anti-angiogenic factors in reducing the tumoral mass [82]. Clinical trials have explored the viability of employing tyrosine kinase inhibitors, such as endothelial growth factor receptor inhibitors (e.g., sorafenib, pazopanib, and sunitinib), in this context [82]. 

### 5.5. Role of Radiologist in Treatment and Management

**Figure 14:**
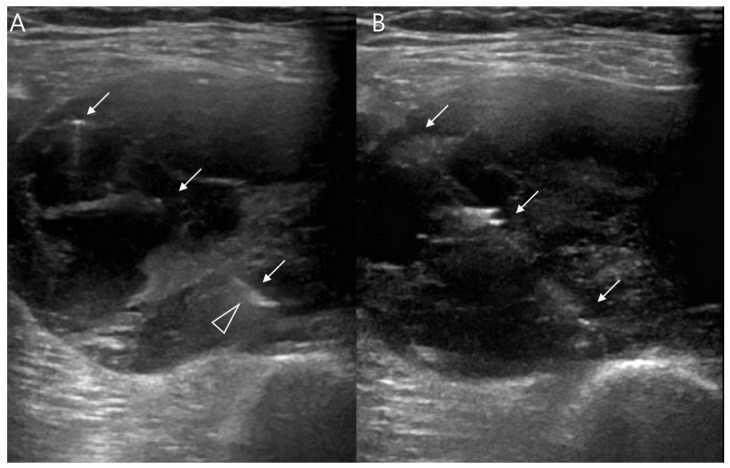
Ultrasound-guided core needle biopsy (same patient with Figure 3): (**A**) Echogenic shaft of biopsy needle (arrows) is relatively well-visualized in the tumor; confirmed that needle tip is within target area (arrowhead). (**B**) Firing of outer cutting cannula is noted to extract tissue specimen (arrows).

A biopsy is essential for an accurate diagnosis in patients with synovial sarcoma to differentiate synovial sarcoma from other soft-tissue sarcoma subtypes and define the tumor grade. Performing a biopsy before the definitive surgery is crucial to prevent inadequate resection and misdiagnosis [83]. To obtain a representative sample of the tumor tissue, there are various options available. These include fine needle aspiration, core needle biopsy, and incisional biopsy [91]. Currently, core needle biopsy with imaging guidance (such as ultrasound or CT for deep, non-palpable masses) is often considered the most suitable choice (Figure 14). A core needle biopsy offers a significant advantage in terms of the quantity of tissue obtained compared to fine needle aspiration, while also presenting a lower risk of complications when compared to an incisional biopsy [92]. The advanced progress in diagnostic imaging has enabled the use of image-guided percutaneous biopsies, leading to enhanced diagnostic performance [93]. Preoperative, multidisciplinary discussions with orthopedic surgeons, radiologists, and pathologists can collaboratively determine the optimal biopsy site and this approach can help minimize the likelihood of obtaining inadequate specimens and enhance the sample quality [94].

**Figure 15:**
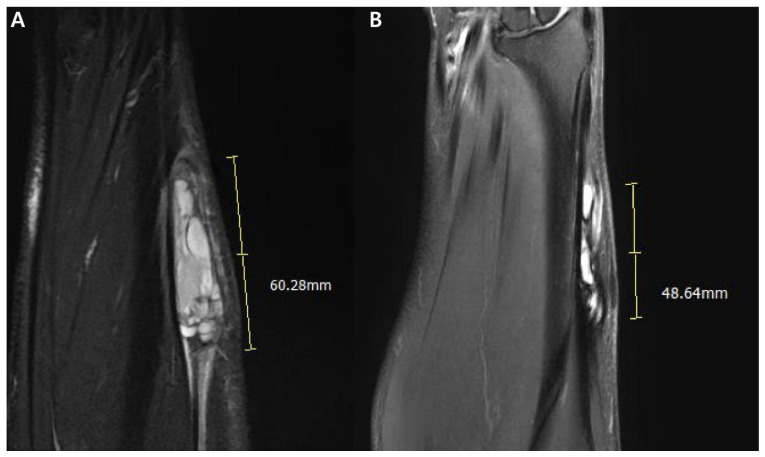
Treatment response after neoadjuvant chemotherapy for synovial sarcoma of 61-year-old-man (monophasic type; same patient with Figure 6): (**A**) Initial MRI of coronal T2-weighted, fat-suppressed image shows about 6 cm sized multilobulated mass with heterogenous signal intensity of Lt forearm showing ‘bowl of grapes’ sign. (**B**) After neoadjuvant chemotherapy, follow-up MRI of coronal T2-weighted, fat-suppressed image shows treatment response with decreased size and decrease of peritumoral edema.

Neoadjuvant chemotherapy and radiation therapy are frequently employed in the treatment of primary synovial sarcomas [38]. MRI can reveal treatment response, often manifesting as a reduction in size (Figure 15). Interestingly, despite positive treatment response, these sarcomas might also experience an increase in size due to necrosis development. Following treatment, subsequent MRI scans are commonly conducted to identify early signs of recurrence. Additionally, routine chest radiographs and CT scans are utilized to detect potential pulmonary metastases [38].

## 6. Conclusions

Synovial sarcomas are malignant tumors that frequently emerge in the extremities of young adults, although they can manifest anywhere across different age groups. This condition exhibits a propensity for late recurrences and the development of metastases. Characterized by an extensive range of imaging presentations, synovial sarcomas encompass both aggressive and non-aggressive features. Recognizing this diverse spectrum, especially the non-aggressive manifestations in smaller sizes, is pivotal for accurate diagnosis, particularly in young adult patients. A soft-tissue mass in a young patient, visible on plain film with calcifications, should consistently raise suspicion for synovial sarcoma. Prominent indicators of larger synovial sarcomas encompass the triple sign, hemorrhage by fluid–fluid levels, and septae revealing a bowl-of-grapes appearance. Certain imaging features serve as predictive markers of adverse outcome, notably larger and more heterogeneous lesions situated in the proximal regions of extremities. Both CT and MRI features play a crucial role in diagnosing synovial sarcomas and predicting prognosis. These imaging findings might be helpful in tailoring of individual patient care. 

## Figures and Tables

**Table 1:** Imaging spectrum of synovial sarcomas.

General imaging features	Periarticular location; Tends to calcify more than other soft tissue tumor (up to 30%).
Various imaging spectrum depending on tumor size	
Small size <5 cm	Non-aggressive features as round, well-defined, homogeneous mass; Almost entirely cystic change as ganglion cyst.
Large size >5 cm	Aggressive features as irregular, ill-defined, heterogeneous mass (internal areas of cystic/necrotic degeneration and hemorrhage) and peritumoral edema; Triple sign and bowl of grapes on T2-weighted images.

**Table 2:** Prognostic factors for synovial sarcomas.

Prognostic Factors		Prognosis
Age	Younger than 15–20 years	Favorable
Tumor size	Large size >5 cm	Poor
Location	Proximal extremity	Poor
	Inguinal region	
	Intrathoracic region	
	Trunk	
Imaging features	Intratumoral hemorrhage (fluid-fluid levels)	Poor
	Triple sign on MRI	
	Bowl-of-grapes appearance on MRI	
	Intercompartment extension	
	Peritumoral enhancement	
	Calcification	Favorable
